# From DNA Methylation Microarray to Digital PCR: A Stepwise Strategy for Tissue Specific cfDNA Biomarker Development

**DOI:** 10.1002/jcla.70210

**Published:** 2026-03-30

**Authors:** David H. Murray, Nicky Boulter, Shanon Ranjit, Natasha M. Rogers, Sarah Kummerfeld, Jason P. Ross, Rodney J. Scott

**Affiliations:** ^1^ Garvan Institute of Medical Research Darlinghurst New South Wales Australia; ^2^ School of Biomedical Sciences and Pharmacy, Faculty of Health and Medicine University of Newcastle Callaghan New South Wales Australia; ^3^ Centre for Transplant and Renal Research Westmead Institute for Medical Research Westmead New South Wales Australia; ^4^ Faculty of Medicine and Health University of Sydney Sydney New South Wales Australia; ^5^ CSIRO Health & Biosecurity Westmead New South Wales Australia; ^6^ Priority Research Centre for Cancer Research, Innovation and Translation Hunter Medical Research Institute New Lambton New South Wales Australia; ^7^ Division of Molecular Medicine, Pathology North John Hunter Hospital New Lambton New South Wales Australia

## Abstract

**Background:**

Tissue‐specific cell‐free DNA (cfDNA) offers promise as a minimally invasive biomarker of organ injury. However, current methods for cfDNA tissue‐of‐origin analysis often depend on genome‐wide sequencing approaches, limiting their clinical scalability.

**Methods:**

We developed a streamlined workflow for translating publicly available DNA methylation datasets into PCR‐compatible biomarker assays, using kidney‐derived cfDNA as a model. DNA methylation microarray data were mined to identify kidney‐specific hypermethylated regions, and candidate biomarkers were screened in silico and experimentally. A digital PCR assay targeting a *PAX2*‐associated DMR was evaluated for analytical performance and assessed in a small exploratory clinical cohort (healthy controls *n* = 9; transplant recipients *n* = 5 pre‐transplant, *n* = 7 at 24 h, *n* = 2 at day 7).

**Results:**

The selected *PAX2* methylation marker demonstrated high analytical specificity for kidney tissue and was not detected in healthy donor plasma or pre‐transplant samples. In contrast, the marker was robustly detected in post‐transplant plasma samples, consistent with acute kidney injury due to ischemia–reperfusion.

**Conclusion:**

This proof‐of‐concept study outlines a scalable method for developing PCR‐based tissue‐specific cfDNA biomarkers. Our kidney‐specific assay demonstrates the potential for rapid, cost‐effective organ injury monitoring and could be adapted for other tissue types. This strategy may support future diagnostic applications in nephrology, oncology, and transplant medicine.

## Graphical abstract

Overview of a DNA methylation biomarker development pipeline, from discovery and assay design to screening, performance evaluation, and specificity testing in clinical sample matrices.

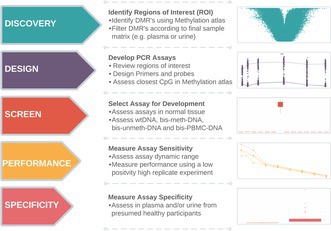

## 1 Introduction

Cell‐free DNA (cfDNA) has emerged as a minimally invasive analyte that enables a broad range of biomarker applications in oncology [1], prenatal screening [2], and transplantation monitoring [3, 4]. In healthy individuals, cfDNA primarily originates from hematopoietic cells, but a significant fraction, estimated at 20%–30%, derives from peripheral tissues through physiological turnover [5, 6]. In pathological states, such as tissue injury or cancer, the release of cfDNA from affected cells increases, offering opportunities for real‐time monitoring of disease processes.

A growing body of research has focused on identifying the tissue of origin (TOO) of cfDNA to improve the specificity of diagnostic tests [5, 7, 8, 9], including kidney injury [10, 11]. This has led to the development of genome‐wide approaches that leverage tissue‐specific DNA methylation signatures. These methods can achieve high‐resolution mapping of cfDNA origins, but often rely on next‐generation sequencing (NGS), which is costly, labor‐intensive, and poorly suited for rapid, point‐of‐care applications.

PCR‐based methods offer a clinically practical alternative, combining ease of validation with high sensitivity, scalability, and rapid turnaround [12, 13, 14]. While such assays typically target a small number of genomic loci, their analytical simplicity and cost‐effectiveness make them attractive for implementation in hospital laboratories. However, the development of methylation‐based PCR assays requires careful biomarker selection and assay optimisation to ensure tissue specificity and compatibility with fragmented cfDNA templates [15].

In this proof‐of‐concept study, we describe a streamlined, data‐driven pipeline for identifying and validating hypermethylated, tissue‐specific markers suitable for PCR‐based detection of cfDNA. Using kidney tissue as a model system, we mined publicly available DNA methylation microarray data to identify differentially methylated regions (DMRs), designed a digital PCR assay targeting the *PAX2* gene, and evaluated its ability to detect kidney‐derived cfDNA in clinical plasma samples. Our goal was to demonstrate that clinically meaningful, tissue‐specific cfDNA markers can be developed without reliance on whole‐genome sequencing or genotyping, and that digital PCR enables sensitive, organ‐specific cfDNA detection for potential use in acute and chronic kidney injury.

This study highlights the feasibility of converting DNA methylation atlas data into robust, low‐cost molecular diagnostics and lays the groundwork for broader application of this approach to other tissues and disease contexts.

## 2 Methods

### 2.1 Methylation Atlas

A Human Methylation Atlas was compiled using DNA methylation data generated on the Illumina Infinium HumanMethylation450 (450 K) or Human MethylationEPIC (EPIC) BeadChip arrays. For all microarray‐derived methylation measurements, we used the Illumina β‐value, defined as the ratio of methylated probe intensity to the sum of methylated and unmethylated probe intensities (β = M/(M + U + 100)), which ranges from 0 (fully unmethylated) to 1 (fully methylated). Data was sourced from The Cancer Genome Atlas (TCGA) and Gene Expression Omnibus (GEO) databases. A total of 1516 samples were collected, including 727 blood samples and 789 solid tissue samples from 21 different tissue sites. Full details of the cohort are included in the Table S2 & S3.

### 2.2 Biomarker Discovery

Kidney‐specific hypermethylation was prioritised during biomarker discovery to enable streamlined primer design and robust multiplexed PCR in downstream assay development. DNA methylation was assessed in the TCGA normal tissue samples (534 samples across 17 tissues) with kidney tissue (45 samples) compared to other tissues (Bile duct, Bladder, Breast, Cervix, Colon, Oesophagus, Head and Neck, Liver, Lung, Pancreas, Prostate, Rectum, Stomach, Thymus, Thyroid gland, and Uterus) to identify kidney‐specific DNA methylation CpG sites. Differential methylation analysis was performed using TCGABiolinks function TCGAanalyze_DMC, which determines the mean DNA methylation difference between each group and uses a Wilcoxon test with the Benjamini‐Hochberg adjustment method to estimate *p*‐values.

CpG sites were categorised as differentially methylated if they (i) had an FDR adjusted *p*‐value less than 1 × 10^−10^, (ii) a differential methylation greater than 0.3 β‐value, (iii) a mean β‐value of less than 0.1 in non‐kidney tissue samples, and (iv) 2 or more differentially methylated CpG probes within a 5 kilobase region. A 5 kb window was used to avoid isolated single‐probe hits and to ensure region‐level methylation suitable for PCR assay design.

These Infinium probes were further filtered using blood DNA cell methylation β‐values from a further set of BeadChip arrays. A total of 716 HumanMethylation450 blood methylation profiles from GEO, including 18 Granulocytes and 36 Peripheral Blood Mononuclear Cells (PBMCs, GSE35069), and 662 Whole Blood samples (GSE40279), were used for this filtering process. Putative DMRs were assessed using the data quantitatively as well as qualitatively, where a positive or negative result was determined using a cutoff β‐value of 0.2. CpG probes with a median β‐value of greater than 0.1 or a sample positivity of over 2% in blood were excluded from further analysis.

To ensure that biomarker selection was not influenced by potential tumour‐adjacent field effects present in TCGA normal kidney samples, we additionally incorporated non‐tumour, histologically normal kidney samples from GEO datasets (see Table S2, S3), including GSE50874 [16]. These healthy and chronic kidney disease (CKD) reference tissues provide an orthogonal validation set to confirm that candidate DMRs reflected true kidney‐specific methylation rather than tumour‐associated alterations. Given TCGA samples were used exclusively for differential methylation analysis, and the GEO originating DNA methylation datasets were used only during the filtering stage (refer to Figure S1), cross‐platform normalisation was not required.

### 2.3 Cases and Controls

Blood was obtained from consented subjects scheduled for kidney transplantation at Westmead Hospital (Sydney, NSW, Australia). Blood was collected prior to transplant and within 24 h and 7 days post‐transplant. The study was approved by the Western Sydney Local Health District Human Research Ethics Committee (2019/ETH02138). Written informed consent was obtained from all participants prior to study enrolment. Plasma from presumed healthy donors were collected by venipuncture under a study approved by the St Vincent's Hospital Human Research Ethics Committee (2021/ETH10901), with informed consent. Commercially available standard material used included hypomethylated DNA sourced from placenta (Merck‐Millipore), fully methylated DNA (Zymo) and PBMC DNA (Roche), which were all bisulphite converted. Native PBMC DNA (i.e., non‐bisulphite converted) was also included.

### 2.4 Sample Collection, Extraction and Bisulphite Conversion

Kidney transplant samples were collected using Streck blood collection tubes and stored for up to 7 days at room temperature. Blood was centrifuged with brakes off at 1600×*g* for 10 min; the plasma layer was removed and underwent another centrifugation at 3000×*g* with brakes off for 10 min. Plasma was stored at −80°C until extraction. Blood from presumed healthy donors was collected by Garvan Research Facility in K_3_EDTA tubes and centrifuged with brakes off at 1500×*g* for 10 min, 22°C within 4 h of collection. Plasma was removed to a new tube and the centrifugation step was repeated. Clarified plasma was then stored in a fresh tube at −80°C until assay.

Cell‐free circulating DNA (cfDNA) was extracted from 1 to 5 mL plasma using the QIAamp Circulating Nucleic Acid Kit (Qiagen, Hilden, Germany) as per protocol. Both cfDNA and tissue DNA (BioChain) were bisulphite converted using the EpiTect Fast Bisulphite Conversion kit (Qiagen).

### 2.5 qPCR and Digital PCR

DNA methylation target regions as well as primer and probe designs are detailed in Table S4 & S5. qPCR was performed on a QuantStudio 7 real‐time PCR system (ThermoFisher) using 1× GoTaq hot start colourless mastermix, 2 mM MgCl_2_, 200 nM of each forward and reverse primers, 100 nM fluorescently labelled hydrolysis probe and template DNA in a final reaction volume of 15 μL, and cycled at 95°C, 2 min [95°C, 15 s; 62°C, 30 s, 72°C, 30 s with acquisition] x 50; 40°C, 10 s. For digital PCR, reactions comprised 10 μL QIAcuity probe PCR mix (Qiagen), 800 nM of each forward and reverse primers, 400 nM fluorescently labelled hydrolysis probe, and template DNA made up to 40 μL final volume, and were cycled at 95°C, 2 min [95°C, 15 s; 61°C, 30 s with acquisition] x 40 on the QIAcuity 4 plate digital PCR system (Qiagen). In most experiments, the input DNA was the equivalent amount from 1 mL of plasma or 5 ng of tissue DNA or control DNA.

### 2.6 Statistical Analysis

All statistical analyses were performed using R version 4.3.2 (R Core Team, Vienna, Austria) [17]. Biomarker discovery was carried out using the TCGAbiolinks (version 2.30.4) and Summarized Experiment (version 1.32.0) R packages. Dimensionality reduction, applied to explore the DNA methylation patterns across tissues, was performed using the base R function prcomp for PCA and the umap package (version 0.2.10.0) for uniform manifold approximation and projection (UMAP). As described in the discovery section, the TCGAanalyze_DMC function was used to identify DMRs while applying the Benjamini‐Hochberg correction to control the false discovery rate (FDR). Descriptive and summary statistics were used where applicable to quantify differences between tissue groups employing an alpha of 0.05.

Data processing and visualisation were completed using standard tidyverse packages, including dplyr (version 1.1.4) and ggplot2 (version 2.4.0.0). For visualisation purposes, DNA methylation β‐values from Illumina microarrays were converted to percentage methylation by multiplying β‐values by 100. All statistical analyses were performed using the original β‐values.

## 3 Results

### 3.1 Overview

**FIGURE 1:**
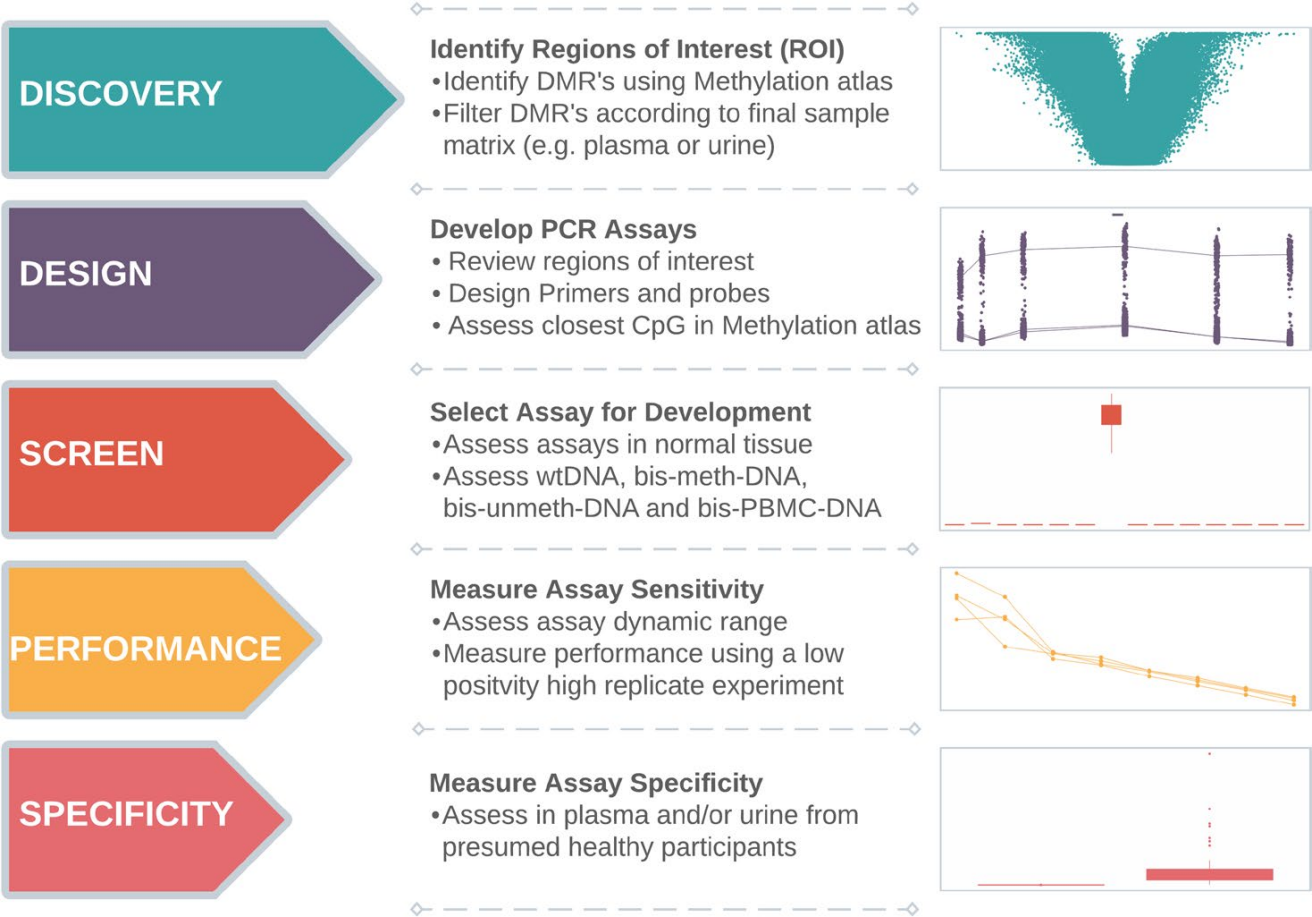
Overview of biomarker development pipeline. Discovery allows identification of biomarkers from DNA methylation data, which can be sourced from public domains or generated internally. PCR designs are prepared and screened using native DNA sourced from Peripheral Blood Mononuclear Cells (PBMCs), referred to as wild type DNA (wtDNA), bisulphite converted methylated DNA (bis‐meth‐DNA), bisulphite converted unmethylated DNA (bis‐unmeth‐DNA) and bisulphite converted Peripheral Blood Mononuclear Cells DNA (bis‐PBMC‐DNA). Performance is measured using bis‐meth‐DNA in custom experimental configurations and specificity is assessed using cfDNA from the sample matrix of choice (e.g., plasma or urine) from presumed healthy individuals.

Building on our previously established assay development strategy [13, 14, 18], we applied a disciplined, industry‐grade biomarker development pipeline consisting of three main phases: discovery, analytical testing, and clinical validation (Figure 1). This rigorous progression prevented premature advancement of weak candidates and guaranteed clinical readiness of selected markers. Selection of a suitable methylation atlas was essential and dependent on the specific clinical or research context. For instance, inclusion of the cancer type of interest is critical when the intended application involves collateral tissue injury detection during cancer treatment due to cancer‐associated methylation changes that may confound tissue‐of‐origin profiles.

### 3.2 Discovery

Methylation biomarker discovery was based on publicly available datasets, including 1516 samples from TCGA and GEO comprising 727 blood and 789 solid tissue samples from 21 tissue sites (Table S2 & S3). Due to missing data in GEO DNA methylation microarrays, initial biomarker selection focused on 534 normal TCGA samples across 17 tissue types. PCA analysis (Figure S2) confirmed tissue‐specific clustering. Kidney‐specific CpG sites were identified using the TCGAanalyze_DMC function [19], comparing kidney tissue (*n* = 45) to all other tissues. Refer to Figure S1 for biomarker selection procedure.

**FIGURE 2:**
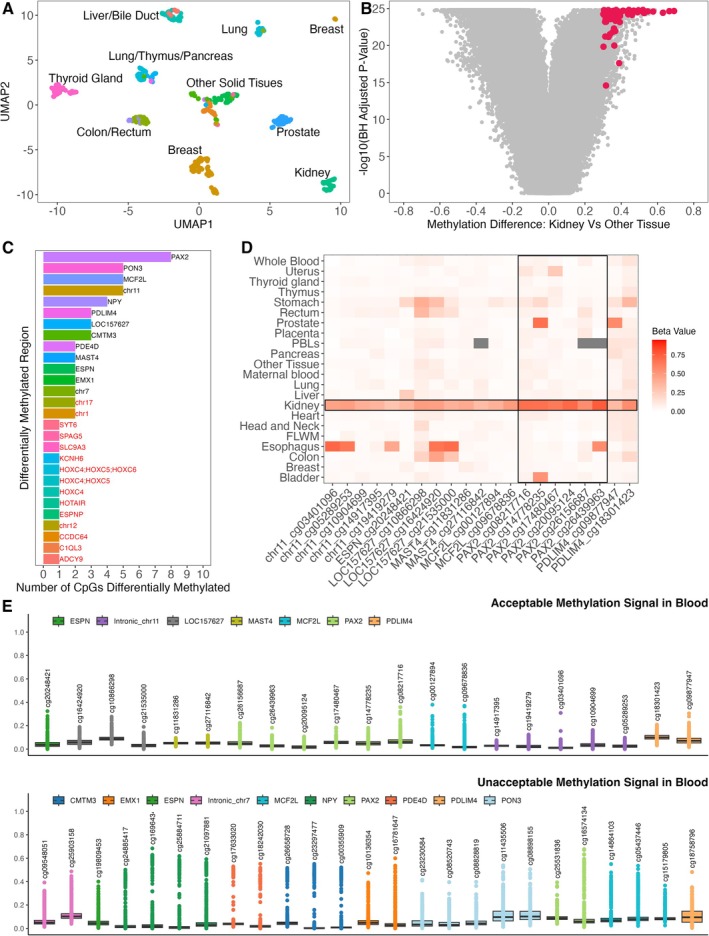
Biomarker discovery for kidney specific cfDNA biomarkers (A). TCGA DNA methylation data assessed using UMAP analysis identified by different colours with significant tissue type clusters labelled (B). Volcano plot showing results of differential methylation analysis for TCGA cohort. Each point represents a CpG site with the mean methylation difference between kidney and other tissues on the x‐axis and an FDR adjust log transformed *p*‐value on the y‐axis. Hypermethylated probes of interest are highlighted using red and were selected based on a β‐value of > 0.3 in kidney tissue relevant to other tissue, < 0.1 β‐value in other tissues and a false discovery rate adjusted *p* < 1 × 10^−10^ (C). For each identified CpG site, we applied a sliding window of 5 kilobases (kb), spanning ±2.5 kb from the CpG site, to assess the regional density of differentially methylated CpG sites. Regions with > 2 CpG probes within this 5 kb window were selected for further assessment are labelled in black. Regions that did not meet this criteria are labelled in red, including regions with 2 probes that are within the 5 kb region, are labelled in red (D). Final probe selection was complete after deprioritising probes with a high methylation signal in blood, refer to Figure 2E. This heatmap shows the mean β‐value of these probes across tissue types from an expanded sample set of 1516 samples (refer to Table S2 & S3 for details). Missing probes are indicated by grey boxes (E). β‐value results for probes from regions selected in figure C assessed in DNA methylation data from whole blood (GSE40279). Probes with a median β‐value of > 0.1 or a sample positivity > 2% when using a β‐value cutoff of 0.2 were not selected for further assessment in Figure 2D.

Tissue‐specific separation varied in the UMAP plot (Figure 2A); for example, liver and bile duct clustered closely, reflecting similar methylation profiles and underscoring the need for stringent, context‐specific thresholds. For kidney tissue, CpGs were considered differentially methylated (Figure 2B) if they met the following criteria: ≥ 0.3 increased methylation β‐value in kidney vs. other tissues, < 0.1 methylation β‐value in other tissues, and FDR‐adjusted *p* < 1 × 10^−10^ (Table S1). Regions with ≥ 2 CpG sites on the BeadChip design within a 5 kb window were retained, yielding 13 DMRs comprising 46 probes (Figure 2C). Although two CpG sites were identified within intronic regions on chromosomes 1 and 17, these probes were separated by more than 5 kb and therefore did not meet the regional density requirement for further assessment.

Blood signal filtering was performed using GEO dataset GSE40279, applying both quantitative (median β‐value < 0.1) and qualitative (< 2% of samples with a β‐value > 0.2) thresholds to eliminate CpGs with background methylation in blood. These stringent criteria ensured minimal off‐target signal and excluded suboptimal candidates. After filtering, 7 DMRs (21 probes) remained (Figure 2D), validated across additional datasets for healthy kidney, brain, placenta, and heart tissue (Figure 2E). Importantly, *PAX2* DNA methylation signal remained high when including the set of 85 micro‐dissected human kidney tubule epithelial samples obtained from GSE50874 [16]. These samples include tissue collected from both healthy individuals and patients with chronic kidney disease.

### 3.3 Design

**FIGURE 3:**
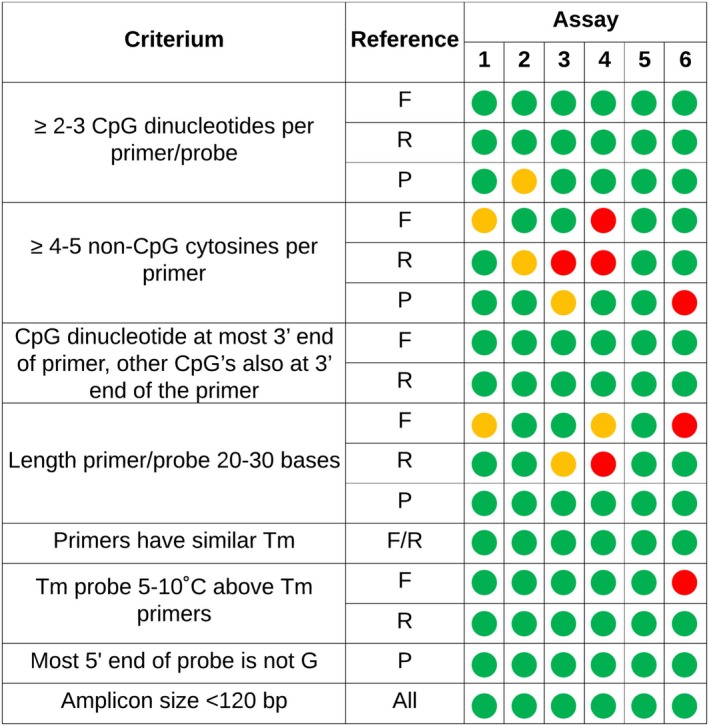
*PAX2* PCR designs (Assays 1–6) assessed according to Masses et al. [15]. Green: Optimal design, yellow: Suboptimal, but acceptable design, red: Increased risk of technical problems with the primer or probe. F: Forward primer, R: Reverse primer, P: Probe.

Methylation‐specific PCR (MSP) assay design followed standard principles with adaptations for cfDNA and bisulphite‐modified targets [15]. Regions with SNVs or indels were avoided. GC content was maintained at 40%–60%, and poly‐G stretches were minimised due to synthesis and probe performance limitations. Following Massen et al. [15], six amplicons (58–72 bp) were designed within the *PAX2* region (Figure 3). Shorter lengths than the 120 bp recommended by Massen were prioritised to accommodate cfDNA fragmentation and urine‐based applications, where shorter fragments predominate as described by Cheng et al. [8]. Primer/probe sequences were designed to span at least two CpGs per oligonucleotide, with the 3′ end of the primer ending on a CpG cytosine to enforce methylation specificity. While a minimum of three non‐CpG cytosines (CH) was preferred across each primer to enforce conversion specificity, this criterion was relaxed to improve design flexibility; off‐target amplification was assessed experimentally.

### 3.4 Screen

**FIGURE 4:**
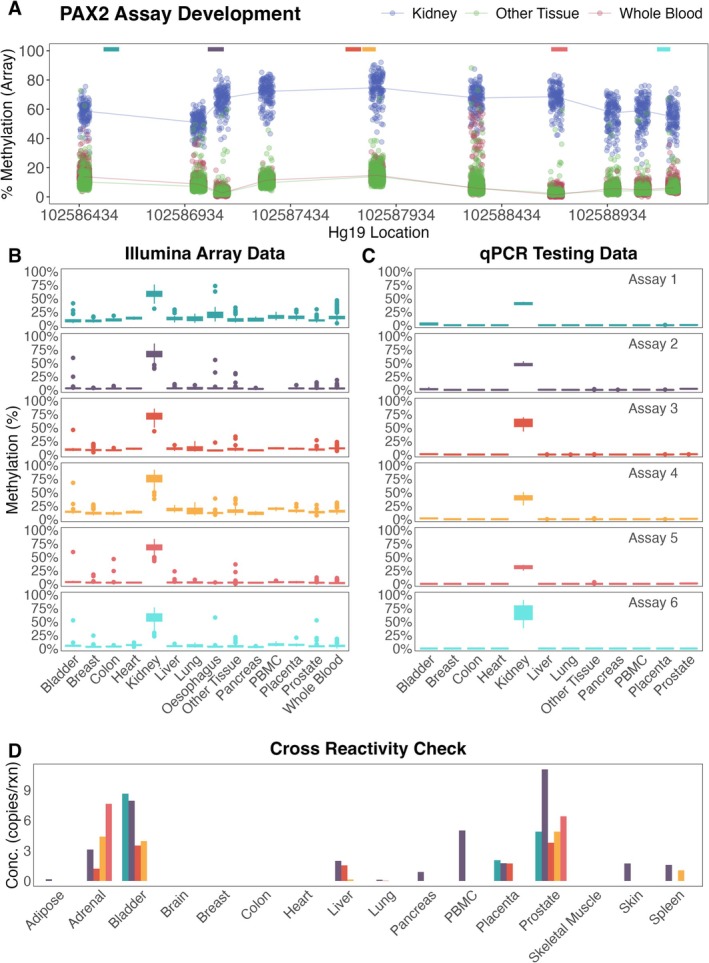
PCR Assay Development (A). Differentially methylated region of interest for *PAX2* shown as % methylation derived from DNA methylation microarray β‐values (converted to % methylation as β × 100) across 1516 samples (refer to Table S2 & S3 for details). Whole Blood and tissue from sites other than kidney are hypomethylated whereas a kidney tissue is hypermethylated. Regions where PCR amplicon were developed is indicated by the coloured boxes, the colours represent the different assay designs as labelled in Figure 4C (B). The % DNA methylation derived from DNA methylation microarray β‐values (β x 100) across 1516 samples for the CpG closest to the PCR target region (C). The % methylation was measured by qPCR assay in 5 ng DNA using the designed *PAX2* assays divided by total DNA measured using *ACTB* as per methods section. From a range of tissue sources. (D) Assessment of cross‐reactivity in all non‐kidney tissue types measured by qPCR for each assay design shown in absolute concentration detected. Kidney values are not shown in this panel to preserve y‐axis resolution for non‐kidney tissues; full data including kidney are provided in (Figure S4).

All six assays were screened via qPCR against a panel including bisulphite‐converted methylated DNA, hypomethylated DNA, wild‐type PBMC DNA, and DNA from 16 tissues. Each reaction included 5 ng input DNA. As shown in Figure 4, most assays demonstrated high specificity for bisulphite‐converted kidney DNA (refer to Figure S3 for assessment in TCGA‐KIRC cohort). Assays 1, 2, and 4 showed the strongest methylation signal in kidney tissue. Assay 5 showed some cross‐reactivity, particularly with bisulphite‐converted adrenal gland DNA. No amplification was observed in unconverted wild‐type DNA, confirming methylation specificity despite relaxed design constraints.

### 3.5 Performance

**FIGURE 5:**
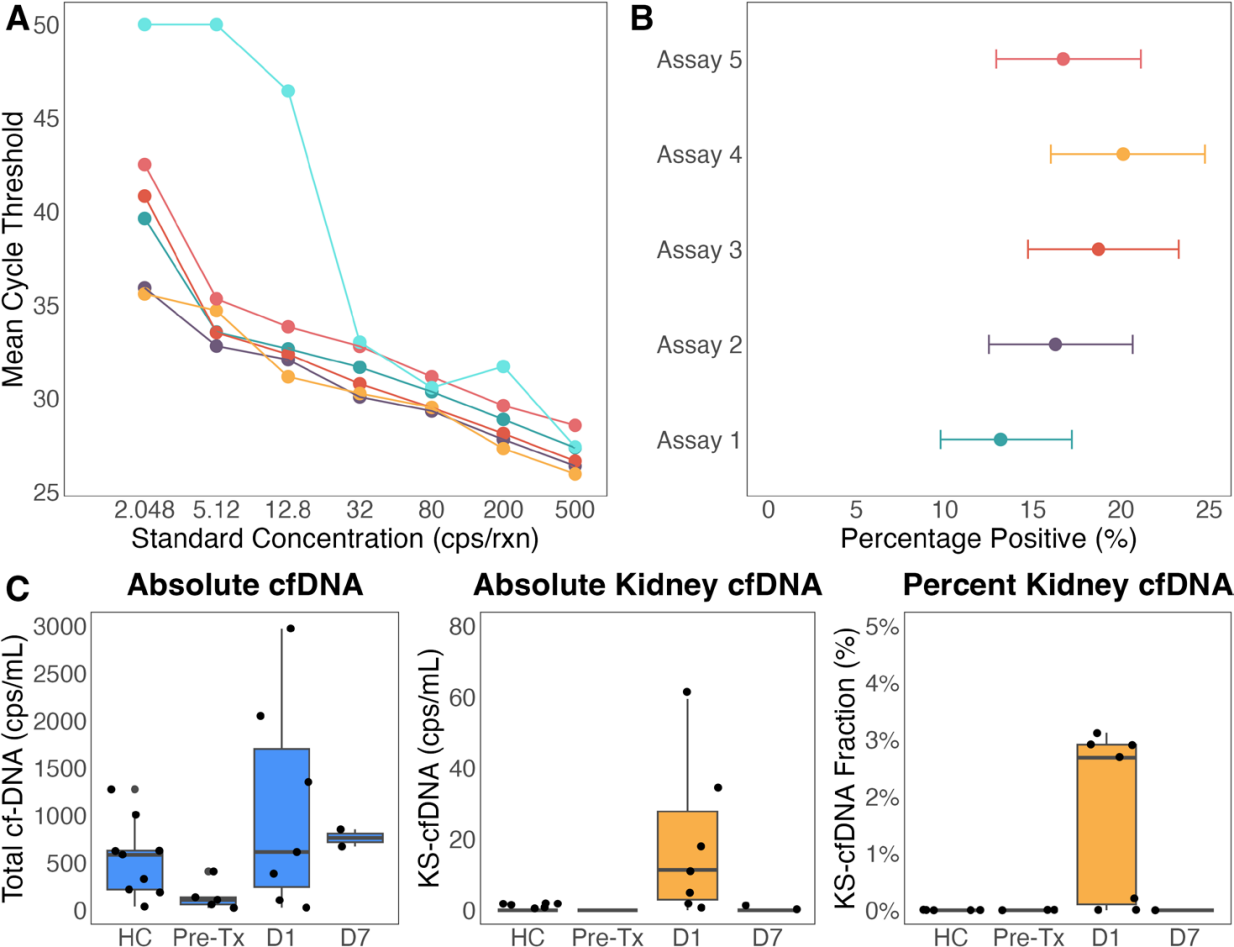
Analytical and Clinical performance evaluation (A). Comparison of standard curve results performed in qPCR between assays to determine assay performance (B). Results from low concentration high replicate experiment to compare assay sensitivity. Assays were tested with between 338–343 replicates each using qPCR (C). Clinical assessment of *PAX2* Assay 4 using digital PCR. Total cfDNA is measured using conversion specific target within *ACTB*. Results are presented using absolute kidney cfDNA and percent of total cfDNA population. HC, Healthy Control; Pre‐Tx, pre‐transplant; D1, day 1 post‐transplant; D7, 7 days post‐transplant.

Assay sensitivity and dynamic range were evaluated using a 7‐point 2.5‐fold dilution series of fully methylated DNA (Zymo), ranging from 500 to 2.048 copies/reaction, tested in eight replicates per point (Figure 5A). Assay 6 failed to meet sensitivity thresholds. To assess single‐copy detection, assays were tested at ~0.17 copies/reaction with > 330 replicates each. Positivity rates were compared using Fisher's exact test. Assay 4 showed the highest sensitivity (20.1%; 95% CI: 16.0%–24.8%), significantly outperforming Assay 1 (13.2%; 95% CI: 9.8%–17.2%, *p* = 0.0181), and was selected for clinical testing (Figure 5B).

### 3.6 Specificity and Clinical Assessment

To assess clinical setting, we evaluated cfDNA detection in a kidney transplant cohort. Acute kidney injury (AKI) resulting from ischemia–reperfusion injury post‐transplant is expected to release kidney‐derived cfDNA into circulation. Plasma was collected from patients before surgery (Pre‐Tx, *n* = 5), and at 24 h (D1, *n* = 7) and 7 days (D7, *n* = 2) post‐transplant. A group of healthy controls (HC, *n* = 9) was also included for comparison.

As shown in Figure 5C, cfDNA concentrations were lower in pre‐transplant end‐stage kidney disease (ESKD) patients than in healthy controls, possibly reflecting underlying illness. While the total cfDNA levels were variable, they showed no statistically significant difference across groups (Kruskal–Wallis *p* = 0.12), indicating the need for kidney‐specific cfDNA biomarkers. In contrast, both absolute and relative measurements of kidney‐specific cfDNA (*PAX2*) demonstrated significant group‐level differences (Kruskal–Wallis *p* = 0.0033 for both). Notably, kidney cfDNA was undetectable in both healthy controls and pre‐transplant patients, but markedly elevated at 24 h post‐transplant, aligning with prior donor derived cfDNA (dd‐cfDNA) findings [20].

## 4 Discussion

Polymerase chain reaction (PCR) is a widely used, cost‐effective technology, well‐suited for hospital laboratory‐based diagnostics. In this study, we present a structured, generalisable method to identify tissue‐specific differentially methylated regions (DMRs), convert them into PCR‐compatible biomarkers, and evaluate their analytical and preliminary clinical performance. Although the method is broadly applicable, we demonstrate its utility through the development of a kidney‐specific cfDNA assay.

Focusing on hypermethylated DMRs, we selected candidate regions with minimal off‐target methylation across other tissues, including blood. Hypermethylated targets offer several advantages: their methylation profiles tend to be more tissue‐specific, and they simplify primer/probe design compared to hypomethylated regions, which often require complex thermocycling conditions and additional conversion‐specific controls, as described by Zemmour et al. [7]. Our designs maximised CpG inclusion within primers and probes and employed a 3′ CpG on the primer to ensure methylation specificity. While multiplexing and sensitivity can be enhanced through the combination of multiple hypermethylated targets, individual assays demonstrate strong specificity, laying the groundwork for future multiplexed panel development.

Many tissues have already been investigated in other studies, including heart [7], breast [21], liver [22] and colon [23]. The use of a kidney transplant cohort provided a valuable proof‐of‐concept context for clinical assessment. Ischemia–reperfusion injury (IRI) following transplantation represents a well‐characterised form of acute kidney injury (AKI) with predictable timing, making it ideal for biomarker evaluation [20]. Using digital PCR, the selected *PAX2*‐based assay reliably detected kidney‐specific cfDNA in plasma following transplantation. Importantly, it was not detected in healthy or pre‐transplant end‐stage kidney disease (ESKD) patients, suggesting a high specificity for injury‐related release. While dd‐cfDNA methods only detect donor‐derived DNA, our assay detects DNA from both native and transplanted kidneys, offering a complementary view of renal damage. Further comparison with dd‐cfDNA is warranted to benchmark this method's clinical relevance and diagnostic resolution. Healthy donor plasma was collected in K_3_EDTA tubes and processed within 4 h, whereas transplant samples were collected into Streck tubes due to clinical workflow requirements. Although Streck tubes minimise leukocyte lysis over extended storage, K_3_EDTA tubes produce comparable cfDNA yields when processed promptly [24]. Thus, differences in tube type are unlikely to explain higher total cfDNA in healthy donors compared with pre‐transplant subjects. Nevertheless, tube‐related effects may contribute modestly to variation in total cfDNA levels, and this is acknowledged as a study limitation.

Beyond transplantation, kidney‐derived cfDNA biomarkers hold potential for broader application in AKI and chronic kidney disease (CKD). While CKD may yield low cfDNA signals due to low epithelial cell turnover, AKI causes rapid and extensive damage, potentially enabling real‐time detection of injury. Current reliance on serum creatinine, a lagging and nonspecific indicator, underscores the need for improved diagnostics. Methylation‐based cfDNA detection could provide earlier and more mechanistically informative insights, particularly when integrated with existing protein biomarkers such as NGAL or KIM‐1 [25].

Incorporating cfDNA biomarkers into clinical decision‐making remains a significant translational hurdle, but the potential gains are considerable. Improved AKI classification and real‐time stratification of injury could enhance care, guide therapy, and facilitate drug development. Our streamlined biomarker development pipeline, beginning with public DNA methylation datasets and ending in a PCR‐compatible assay, offers a scalable alternative to genome‐wide sequencing approaches. While preliminary, this work serves as a foundation for future studies across multiple tissues and disease contexts.

Ultimately, this proof‐of‐concept demonstrates the feasibility of developing tissue‐specific methylation biomarkers for cfDNA detection using digital PCR. Our results support the clinical utility of *PAX2*‐based assays for specific detection of kidney injury; however, given the small size of our transplant patient cohort these findings should be interpreted cautiously. The main limitation of this study is the small clinical sample size, particularly at the day‐7 timepoint, which reflects the exploratory nature of this analysis. While the observed increase in kidney‐derived cfDNA is biologically consistent and technically robust, larger studies with standardised sample collection will be required to define clinical sensitivity, specificity, and temporal kinetics. Future studies should focus on expanding sample sizes and evaluating the assay in diverse clinical contexts to support broader clinical translation. Continued refinement, validation, and integration with clinical workflows will be essential to realising the full potential of this approach across nephrology and beyond.

## Author Contributions

D.H.M., N.B., and N.M.R. coordinated the study. D.H.M., N.M.R., N.B., and S.R. were responsible for the data collection. D.H.M. was responsible for the data analysis and interpretation of results. D.H.M. drafted the manuscript. All authors read and approved the final manuscript to be published.

## Funding

Funding was obtained from the Kinghorn Foundation and reagent contributions from Qiagen.

## Conflicts of Interest

David Hugh Murray (DHM) and Nicola Boulter (NB) are listed as inventors on a patent application filed by the Garvan Institute of Medical Research related to aspects of this work (International Application No. PCT/AU2023/050562). The remaining authors declare no competing interests.

## Supporting information


**Figure S1:** Flowchart of decision maker in biomarker identification.
**Figure S2:** PCA plot for TCGA normal tissue data.
**Figure S3:** PAX2 Region in Normal Samples (*n* = 160) from TCGA KIRC cohort.
**Figure S4:** Cross‐reactivity analysis including kidney tissue.
**Table S1:** List of CpG probes identified in Differential Methylation Analysis.
**Table S2:** Overview of normal tissue data sourced from TCGA database.
**Table S3:** Overview of normal tissue data sourced from Gene Expression Omnibus (GEO) database.
**Table S4:** Summary of PAX2 targets with target locations recorded using GRCh37/hg19 as per Illumina methylation array manifest files.
**Table S5:** Primer and probe designs for PAX2 targets.

**Data S1:** Supporting Information.

## Data Availability

The datasets analysed during this study are available from the corresponding author on reasonable request. All publicly sourced data has been listed in Tables S2 and S3.
