# Supplementary material for: From DNA Methylation Microarray to Digital PCR: A Stepwise Strategy for Tissue Specific cfDNA Biomarker Development
Source: J Clin Lab Anal. 2026 Mar 30;40(9):e70210. doi: 10.1002/jcla.70210 (PMC13163929; doi:10.1002/jcla.70210)
Supplement: Supplementary file 1 — Figure S1: Flowchart of decision maker in biomarker identification. Figure S2: PCA plot for TCGA normal tissue data. Figure S3: PAX2 Region in Normal Samples (n = 160) from TCGA KIRC cohort. Figure S4: Cross‐reactivity analysis including kidney tissue. Table S1: List of CpG probes identified in Differential Methylation Analysis. Table S2: Overview of normal tissue data sourced from TCGA database. Table S3: Overview of normal tissue data sourced from Gene Expression Omnibus (GEO) database. Table S4: Summary of PAX2 targets with target locations recorded using GRCh37/hg19 as per Illumina methylation array manifest files. Table S5: Primer and probe designs for PAX2 targets. [file JCLA-40-e70210-s001.docx]

**From DNA Methylation Microarray to Digital PCR: A Stepwise Strategy for Tissue Specific cfDNA Biomarker Development**

**
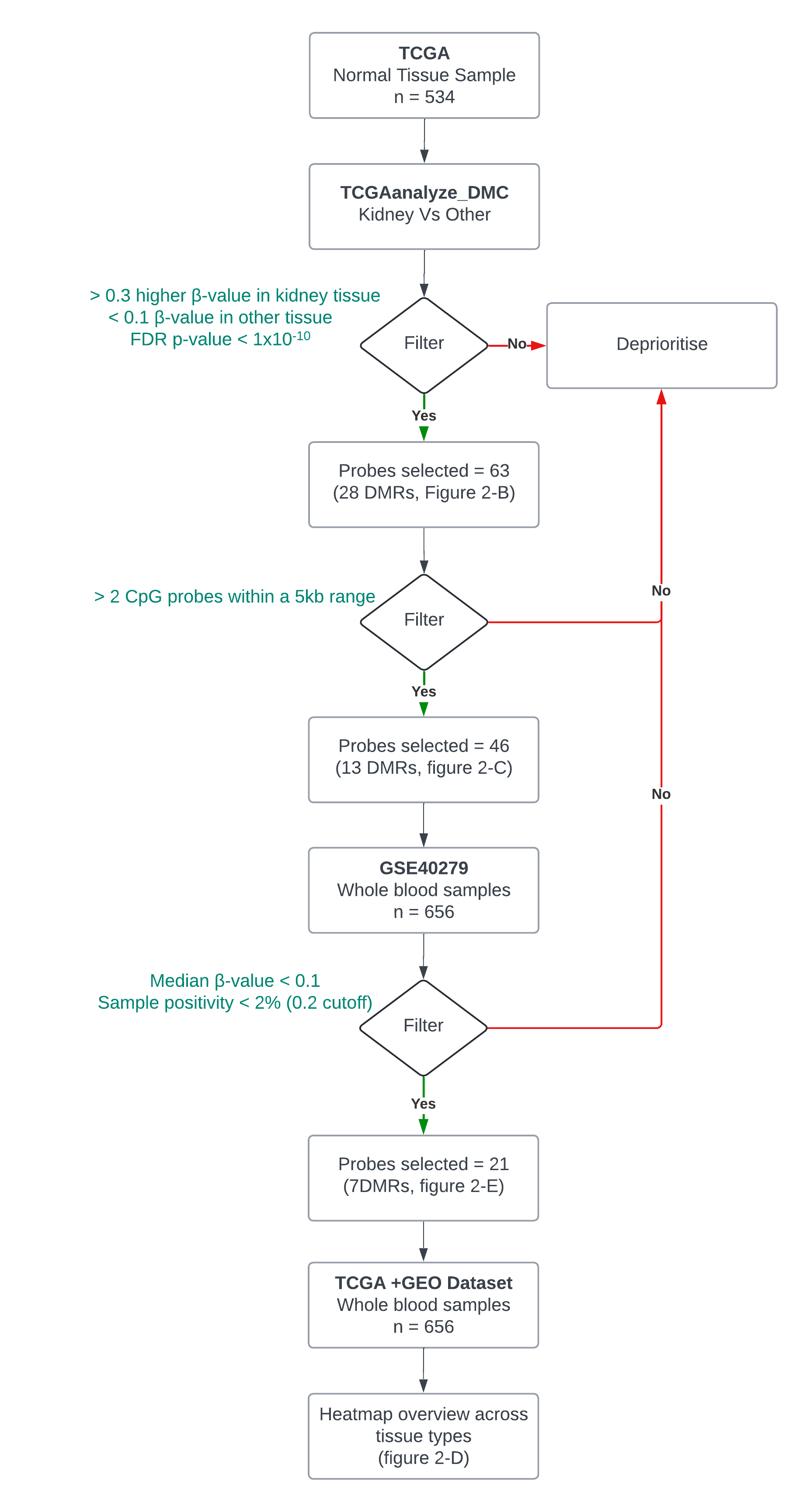
**

**Supplementary Figure 1:** Flowchart of decision maker in biomarker identification.


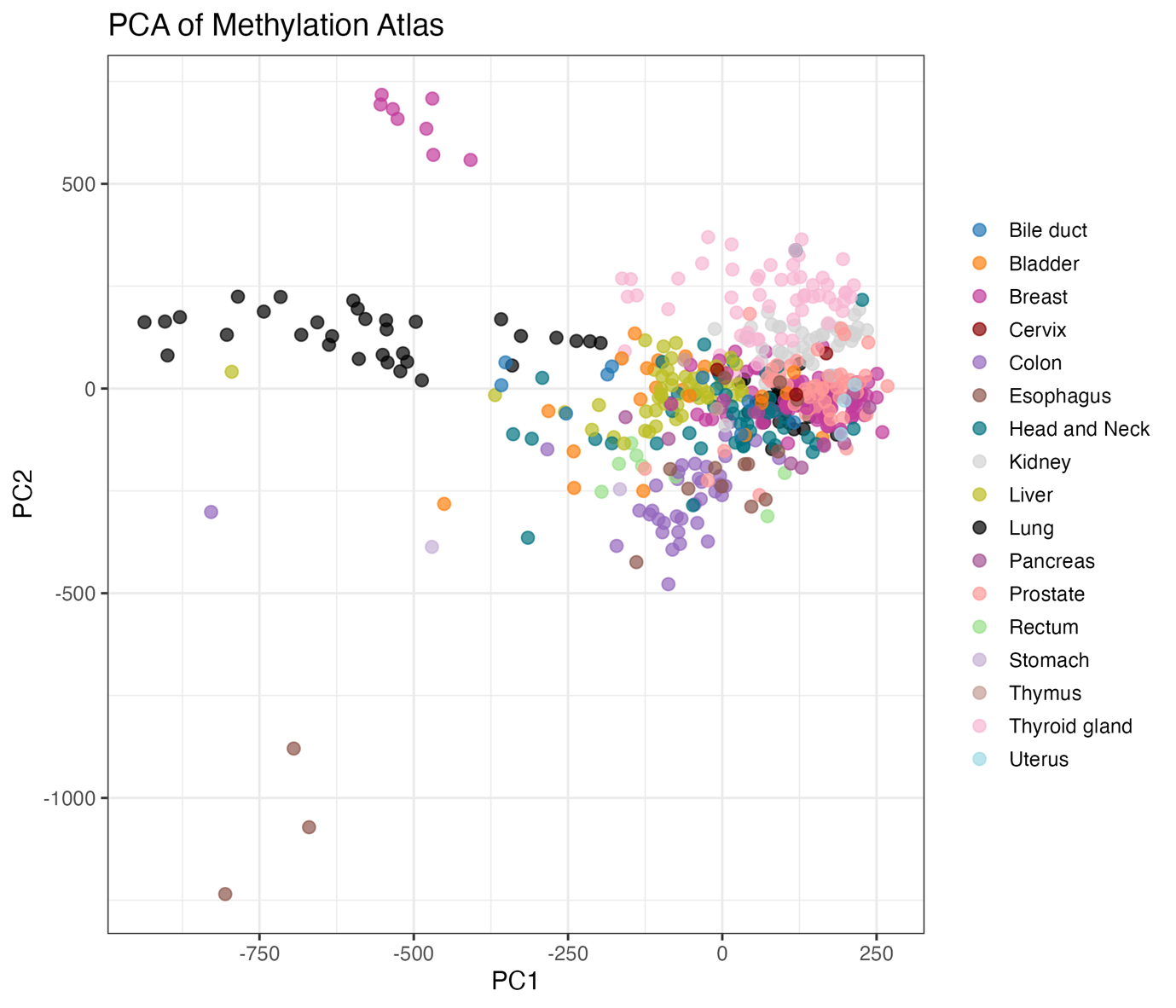


**Supplementary Figure 2:** PCA plot for TCGA normal tissue data

The plot represents a Principal Component Analysis (PCA) of the Methylation Atlas, displaying the variation in methylation profiles across various tissue types. Each point corresponds to an individual sample, with different colours indicating tissue types as detailed in the legend. The first two principal components (PC1 and PC2) capture the primary axes of variation, with PC1 on the x-axis and PC2 on the y-axis. Samples cluster according to their tissue of origin, demonstrating distinct methylation profiles across tissues. While a few outliers are present, they were not removed from the analysis, as they may represent biologically relevant variability, such as rare tissue subtypes or natural methylation differences. Retaining these outliers ensures that the full range of biological diversity is captured in the analysis, avoiding potential loss of important insights.


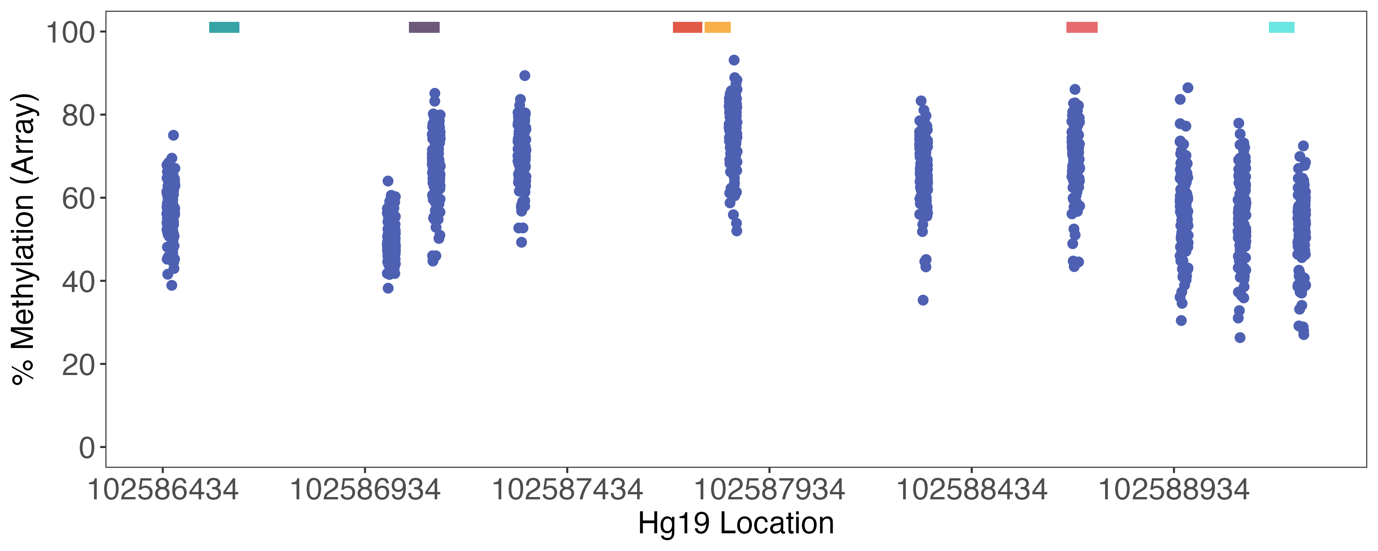


**Supplementary Figure 3:** PAX2 Region in Normal Samples (n = 160) from TCGA KIRC cohort.

DNA methylation profiles across the *PAX2* differentially methylated region (DMR) are shown as percentage methylation, derived from DNA methylation microarray β-values (converted to % methylation as β × 100). TCGA KIRC normal samples were not included in the original methylation atlas used for biomarker discovery. For completeness, the *PAX2* biomarker was analysed separately in this cohort, demonstrating consistent methylation patterns with those observed in KIRP normals.


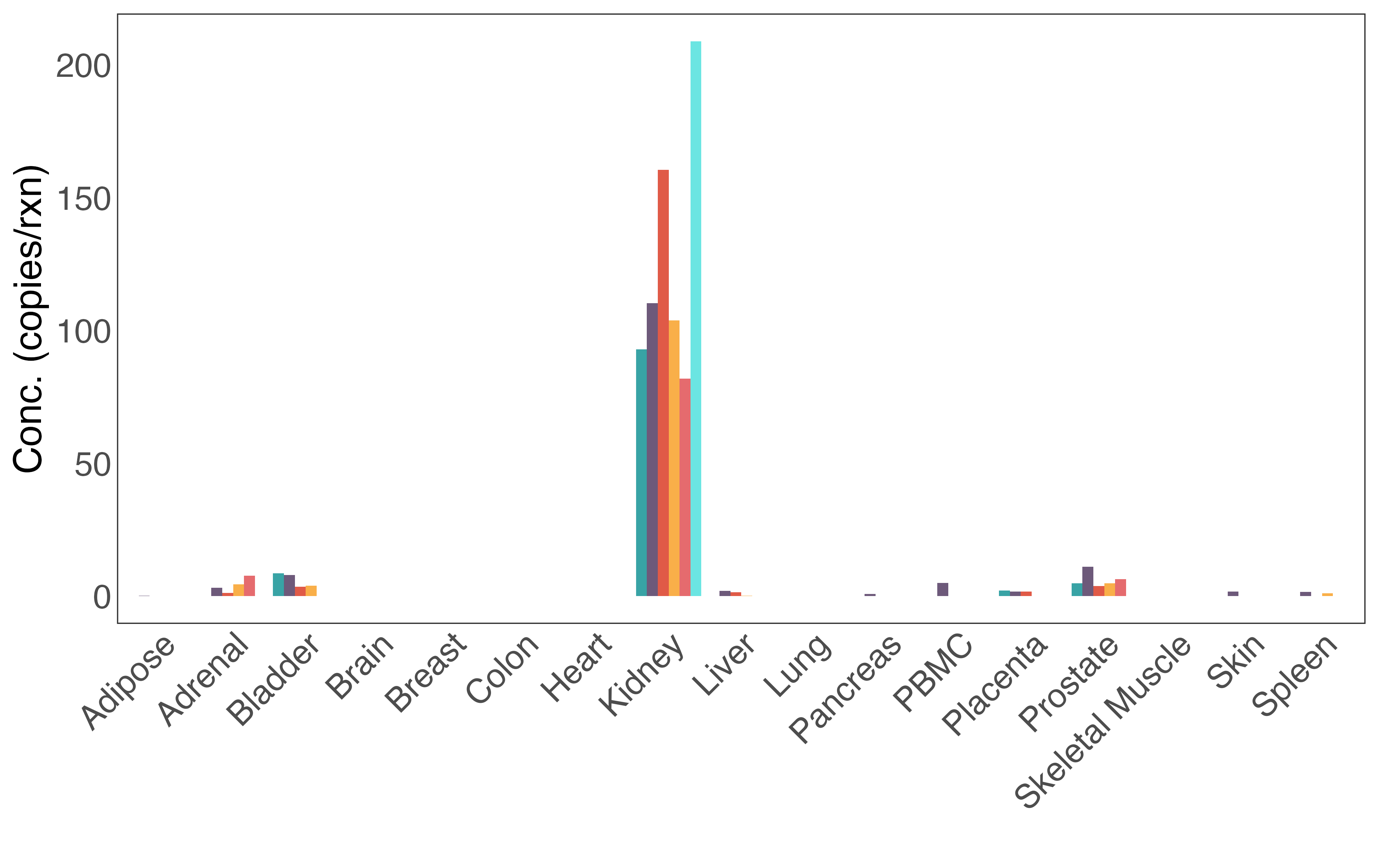


**Supplementary Figure 4:** Cross-reactivity analysis including kidney tissue.

This plot is a representative of the figure shown in Figure 4D form the main paper with the exception that kidney is included for comparison. Absolute concentrations (copies/mL) measured for each assay are shown across tissues on an expanded scale to provide reference values while preserving interpretability.

**Supplementary Table 1:** List of CpG probes identified in Differential Methylation Analysis.

**Supplementary Table 2:** Overview of normal tissue data sourced from TCGA database.

**Supplementary Table 3:** Overview of normal tissue data sourced from Gene Expression Omnibus (GEO) database.

**Supplementary Table 4:** Summary of PAX2 targets with target locations recorded using GRCh37/hg19 as per Illumina methylation array manifest files.

**Supplementary Table 5:** Primer and probe designs for PAX2 targets.
